# Outcomes of Finger Replantation in Western Nepal: An Observational Study

**DOI:** 10.31729/jnma.9121

**Published:** 2025-06-30

**Authors:** Piyush Giri, Sujan Shakya

**Affiliations:** 1 Department of Surgery, Pokhara Academy of Health Sciences, Pokhara, Kaski, Gandaki, Nepal; 2 Department of Orthopaedics, United Mission Hospital Tansen, Palpa, Nepal

**Keywords:** *finger replantation*, *fingertip replantation*, *survival rate*, *functional outcome*

## Abstract

**Introduction:**

Hand injuries involving finger amputations prevalent in young working populations. This study evaluated the demographics and clinical outcomes of finger replantation procedures performed at a regional referral center in Western Nepal.

**Methods:**

This retrospective,observational, cross-sectional study was conducted at the Department of Burns, Plastic and Reconstructive Surgery, Charak Memorial Hospital, Pokhara, from January 2023 to December 2024. Ethical approval was obtained from National Health Research Council on 16 February 2025 (Reference no:1840). All patients who underwent finger replantation procedure with successful artery and venous flow restoration were included. Data on demographics, injury characteristics, surgical techniques, and outcomes were collected. Survival was defined as digit viability for a minimum of 21 days. Functional outcomes were assessed using the Chen functional scoring system at 6 months.

**Results:**

A total of 10 finger replantation procedures were performed in 10 patients with mean age of 34.6 ±13.60 years. All patients were male and right-handed. Six (60%) patients were employed in blue-collar occupations. The thumb was commonly affected digit accounting for 6 (60%) cases. The principal mechanism of injury was crush amputation observed in 7 (70%) cases. Five (50%) replanted fingers were categorized as Tamai level III. Successful replantation was achieved in 8 (80%) fingers. Functional assessment revealed Grade I outcomes in 6 (75%) cases and Grade III in 2 (25%) cases.

**Conclusions:**

Finger replantation in a resource-limited setting achieved survival rates comparable to international standards. Success was attributed to younger patient age, shorter ischemia time, and appropriate surgical techniques.

## INTRODUCTION

Replantation of a finger is a technically challenging microsurgical procedure. The primary objective of replantation is to restore blood flow and facilitate functional recovery. Successful replantation requires meticulous surgical planning, advanced microsurgical skills, and comprehensive postoperative care, with decisions influenced by patient factors, cultural values, and surgeon expertise ^1-4^

Finger amputation is common among young and middle-aged adults, particularly those engaged in machinery-related occupations. Finger amputations are especially debilitating, leading to severe functional and psychological consequences, particularly in younger populations.^5^

The milestone achievements of Ronald Malt's arm replantation in 1962 and Komatsu and Tamai's finger replantation in 1965 established the foundation for modern microsurgical approaches.^6,7^ Despite technological advances, successful outcomes remain challenging in resource-limited settings where specialized equipment, trained personnel, and postoperative care may be constrained.

This study evaluates clinical characteristics, survival rates, and functional outcomes in patients undergoing finger replantation at a regional center in Western Nepal.

## METHODS

This is an observational cross-section study conducted at the Department of Burns, Plastic and Reconstructive Surgery, Charak Memorial Hospital, Pokhara, Gandaki, Nepal. The hospital serves as a regional referral center for complex reconstructive procedures and is one of the few centers in Nepal equipped with facilities for microsurgical procedures. This positioning makes it ideal for examining microsurgical outcomes in a rural healthcare context, where access to specialized care is limited.

Ethical approval for the study was obtained from ethical approval board of National Health Research Council dated 16^th^ February, 2025 (Reference number:1840). The study included data of patient who underwent finger replantation between January 1, 2023 and December 31, 2024. All patients who underwent finger replantation and fit were considered study sample. The inclusion criteria included all patients with finger amputation who underwent replantation surgery with successful completion, defined as the establishment of both arterial perfusion and venous outflow to the replanted digit at the conclusion of surgery. Patients with finger amputations who did not undergo replantation were excluded.

Data were extracted from medical records using a structured proforma designed specifically for this study. Patient's age, sex, occupation, hand dominance was recorded. Injury characteristics, including mechanism of injury (guillotine, crush, avulsion, or combination), number of fingers involved, amputation level according to theTamai classification as described by Tamai (Table 1), time from injury to replantation, ischemia time, and hospital transportation mode were also recorded.^8^

In this retrospective study, the method of transporting amputated digits was reviewed. Appropriate transport was defined as wrapping the digit in moist gauze, placing it in a plastic bag, and storing it in a container with ice.

**Table 1:** Tamai Classification of Digit Amputation Level Description

Level	Description
I	Distal to FDP insertion
II	Distal interphalangeal joint to FDP insertion
III	Middle phalanx distal to FDS insertion
IV	Proximal phalanx distal to FDS insertion
V	Metacarpophalangeal joint and proximal

FDP=Flexor Digitorum Profundus; FDS=Flexor Digitorum Superficialis

**Table 2:** Chen Functional Scoring System

Grade	Return to Work	Range of Motion	Sensory Recovery	Motor Recovery
I	Resume original job	> 60% of normal	Normal/Near Normal	Grade 4/5
II	Resume suitable work	>40% of normal	Near Normal	Grade 3/4
III	Activities of daily life	>30% of normal	Partial recovery	Grade 3
IV	Almost no function			

**Table 3:** Clinical profile of patients with finger replantation procedures (n=10).

Category	n(%)
Age Groups	
15-25 years	2(20)
26-40 years	4(40)
41-58 years	4(40)
Gender	
Male	10(100)
Female	0(0)
Hand Laterality	
Right	10(100)
Left	0(0)
Occupation	
White collar	1(10)
Blue collar	6(60)
Student	3(30)
Smoking Status	
Present	4(40)
Absent	6(60)
Replanted Fingers	
Thumb	6(60)
Index	0(0)
Long	2(20)
Ring	2(20)
Small	0(0)
Mechanism of Injury	
Crush amputation	7(70)
Sharp amputation	3(30)
Transport Method	
Adequate	3(30)
Inadequate	7(70)
Amputation Level (Tamai)	
Level I	1(10)
Level II	1(10)
Level III	5(50)
Level IV	3(30)
Level V	0(0)

**Table 4:** Survival Rate of Finger Replantation

Outcome	n(%)
Total replanted fingers	10(100)
Successful replantation	8(80)
K-wire insertion	8(80)
Failed replantation	2(20)

**Table 5:** Functional Outcomes Using Chen Scoring System

Chen Grade	n(%)
Grade I	6(75)
Grade II	0(0)
Grade III	2(25)
Grade IV	0(0)

Records were evaluated to determine whether amputated fingers were brought within 12 hours (warm ischemia) or 24 hours (cold ischemia), which was considered adequate preservation. Surgical details extracted included the type of replantation procedure, microsurgical techniques utilized, number of arteries, veins, and nerves repaired, additional procedures performed, and postoperative anticoagulation protocols. Documentation of procedural complications and their management was also analyzed.

The survival of the replanted digit was assessed based on viability for a minimum of 21 days. Functional outcomes were evaluated at 6 months postoperatively using the Chen functional scoring system. (Table 2).^9^

All data analysis were conducted using Microsoft Excel Version 16.45. Descriptive statistical analyses were conducted, with the findings presented in tabular form. Categorical variables are reported as frequencies and percentages, whereas continuous variables are expressed as means with standard deviations. This retrospective study employed de-identified data from existing medical records, thereby posing minimal risk to participants. The study protocol conformed to institutional guidelines for retrospective research and medical record review.

## RESULTS

During the study period, 10 patients underwent finger replantation procedures, with a total of 10 replanted fingers. The mean age was 34.6±13.6 years (range: 15-58 years). All patients were male and right handed (Table 3).

The average time of presentation was 4.00 ±1.73 hours (range: 1-8 hours). Three (30%) of the amputated fingers were transported using adequate preservation methods. Crush amputation,while cleaning bicycle chains accounted for 7 (70%) cases and sharp amputation for 3 (30%) cases. Tamai level III amputation was noted in 5 (50%) cases and level IV amputation in 3 (30%) cases.

Arterial repair was performed in each case, with the dominant digital artery being repaired in 7(70.00%) cases. For proximal amputations classified as Tamai level II, anastomosis was performed proximal to the central arterioles. Venous repair was successful in 8 (80%) cases, with a preference for repairing the dorsal vein in 7 (87.5%) cases. Bone fixation was achieved using double Kirschner wires in 8 (80%) cases. Auxiliary procedures were required in 5 (50%) cases, including local flap coverage in 4 (40%) cases and vessel grafting in 1 (10%) case.

Of the 10 replanted fingers, 8 (80%) were successfully reattached (Table 4). Physiotherapy and occupational therapy began on the fifth post-operative day in 7 (70%) of patients and on second post-operative day in 3 (30%) patients.

Functional outcomes were assessed using the Chen functional scoring system at 6 months postoperatively (Table 5). Of the 8 successfully replanted fingers, 6 (75%) cases achieved a Grade I functional outcome, indicating excellent function (Table 5). The two (25%) patients with lower functional outcomes had Tamai level III amputations of the index and long fingers.

## DISCUSSION

Replantation is defined as the restoration of a completely amputated body part. Optimal outcomes after replantation depend on functional and aesthetic results, as well as successful microvascular anastomosis.^10^ In this study, the success rate was 80% (8 successful replantation out of 10 total procedures), which is comparable to studies from Asian countries reporting rates of 85% to 100% with higher success rate in high volume centers. Altam et al. (2024) analyzed 21 microsurgical replants in similar resource limited setting over 7 years, achieving a 76.2% success rate. Significant predictors of failure included multiple digits, complete avulsion, and comorbidities like smoking, diabetes, or hypertension. Success rates vary considerably in the literature, with studies reporting survival rates ranging from 60% to 94%, and generally lower rates observed in children (69.30%) than in adults (76.30%).^5^, ^11-13^

Fejani et al. in their meta-analysis to determine predicting factors for successful replantation found that the early replantation group (<6 hours) had 40% greater odds of survival compared to the 6-12 hours replantation group. Additionally, other predictors for higher survival rates included clean-cut amputation, increased number of primary venous and arterial anastomoses, and non-smoking status.^14^

The favorable survival rate in our study can be attributed to several factors: proximal finger amputations, shorter ischemia times (mean 4 hours, range 1-8 hours), adequate transportation methods in 30% of cases, fewer smokers (40% patients), younger age group involvement (mean age 25.4 years), and absence of comorbid conditions affecting peripheral circulation.

In this study all patient were male, working in manual labor (60% blue-collar workers), with the thumb being the most frequently affected digit (60% of replanted digits), consistent with the results of epidemiology study in brazil. Mattar et al found that thumb replantation was the most frequently performed procedure, predominantly affecting working-age men engaged in physically demanding occupations.^15^

The literature review revealed varying success rates depending on the injury mechanism, with higher survival rates typically observed for non-crush compared to crush amputations. In this study, most replanted fingers (70%) resulted from crush injuries, particularly sustained while cleaning motorcycle chains; however, success rates remained comparable to those of other studies in similar resource limited setting in Yemen with crush amputation being most common mechanism in 15 of 21 fingers analyzed in the study , suggesting that favorable outcomes can be achieved with appropriate surgical technique and postoperative management.^12,16,17^

In this study, the most replanted fingers were thumbs (60%), with Tamai zone III being the most common amputation level (50%). Amputation of thumb is absolute indication for replantation regardless of level due to it critical function for grip and opposition which constitute almost half of function of the hand.^18^ Patient beliefs in Asian countries differ from those in Western countries. In our region, which aligns with Confucian moral values, there is greater emphasis on maintaining body integrity. Although literature indications are well established, we believe that strong patient desire is also a significant factor in replantation decisions. Except for definite surgical contraindications, patients' wishes are considered when making treatment decisions.^19, 20^

Some literatures emphasize that the justification for single finger replantation hinges on the replanted digit achieving adequate sensory and motor function. ^13,21^ In contrast, Waikakul et al. found that all 237 patients who underwent finger replantation reported satisfaction with the procedure, even when some experienced less than optimal function, highlighting the role of cultural influences in decisions about digit replantation.^17^ From a medico economic perspective, single-digit replantation has a higher incremental cost- effectiveness ratio than revision amputation.^22^

In this study, the majority of the replanted fingers were stabilized using two Kirschner wires for bone fixation. During the early postoperative period, one patient experienced a fracture, indicating a lower complication rate compared to the findings of Cho et al., who reported bony issues in replanted fingers in 30-50% of cases, with nonunion rates of 10-30% and malunion rates of approximately 20%.^21^

In terms of venous repair, 60% of the replanted fingers underwent single-vein repair, 20% had two venous anastomoses, and 20% had no venous repair. Various studies have shown that venous anastomoses affect survival rates, with multiple venous anastomoses linked to higher success rates. However, achieving multiple venous anastomoses is not always technically feasible in distal replantations.^11,23^ In most cases (87.50%), venous anastomosis was performed dorsally, while only 12.50% had palmar venous anastomosis. The digital veins distal to the distal interphalangeal joint are generally larger on the palmar side than on the dorsal side, with the opposite being true proximally.^24^

In this study, we repaired only one artery in all the replanted fingers, favoring the dominant digital artery at the proximal level. Ono et al. indicated that repairing only one digital artery is adequate for successful replantation. In the thumb, index, long, and ring fingers, the ulnar digital artery is dominant, whereas in the small finger, the radial digital artery takes precedence.^1^

Nerve recovery is crucial for enhancing postoperative functional outcomes. In our study, the repair of both digital nerves contributed to improved functional results, as demonstrated in other research. Factors positively influencing sensory recovery and functional outcomes included a younger age group, absence of comorbid conditions, short ischemia time, and early rehabilitation, as reported in similar studies.^3,16^

Conversely, the two patients with poorer functional outcomes in this study had amputations at Tamai level III, affecting the index and long fingers. These suboptimal outcomes can be attributed to several factors including prolonged immobilization and poor compliance with postoperative rehabilitation, which resulted in joint stiffness and limited range of motion. The level of injury appears to be a significant predictor of functional outcomes, with joint-level amputations demonstrating worse functional scores compared to phalanx amputations, likely due to increased stiffness and decreased range of motion resulting from joint involvement. Additionally, the mechanism of injury influences outcomes, with crush and avulsion injuries associated with poorer functional results due to the severity of trauma and subsequent inflammation of soft tissue structures including tendons, flexor sheaths, and ligaments, which may impair normal digit function and mobility.^25^

The most common postoperative complication was venous insufficiency (30%), followed by arterial complications (10%), aligning with other published studies. Two replantation failures were thumb and ring finger following crush amputation and due to venous complication. Rao V et al. noted that alterations in Virchow's triad are the mechanism behind venous thrombosis, typically occurring within the first three postoperative days, which is consistent with our findings. The main prognostic factors for success include surgical technique, injury type, and injury mechanism, with postoperative antithrombotic regimens being crucial for preventing thrombosis.^23^ This is particularly relevant in cases of crush or traction injuries, when vessel grafts are used (10% of our cases), or when intraoperative findings suggest an increased risk of thrombosis. However, these measures cannot replace properly performed anastomoses.

This small number reflects the rarity of finger replantation procedures in our rural setting. This study addresses a significant gap in regional literature by reporting finger replantation outcomes from Western Nepal, where published microsurgical data is limited. Cultural considerations unique to this region, where traditional beliefs about body integrity influence replantation decisions differently from Western practices, add important context to surgical decisionmaking and highlight the need for region-specific outcome data to guide clinical practice in similar settings.^26^

## CONCLUSIONS

The authors would like to express their sincere gratitude to the Department of Burns, Plastic and Reconstructive Surgery, Charak Memorial Hospital for providing clinical support and logistical resources.
